# Clinical characteristics and outcomes of methamphetamine-associated versus non-methamphetamine intracerebral hemorrhage

**DOI:** 10.1038/s41598-020-63480-z

**Published:** 2020-04-14

**Authors:** Zhu Zhu, Sahar Osman, Dana Stradling, Mohammad Shafie, Wengui Yu

**Affiliations:** 1 Department of Neurology, University of California, Irvine, CA USA; 2 Department of Neurology, Huashan Hospital, Fudan University, Shanghai, China

**Keywords:** Risk factors, Stroke

## Abstract

Methamphetamine use has emerged as a risk factor for intracerebral hemorrhage (ICH). We aim to investigate the clinical characteristics and outcomes of methamphetamine-associated ICH (Meth-ICH) versus Non-Meth-ICH. Patients with ICH between January 2011 and December 2017 were studied. Meth-ICH and Non-Meth-ICH were defined by history of abuse and urine drug screen (UDS). The clinical features of the 2 groups were explored. Among the 677 consecutive patients, 61 (9.0%) were identified as Meth-ICH and 350 as Non-Meth ICH. Meth-ICH was more common in Hispanics (14.6%) and Whites (10.1%) as compared to Asians (1.2%). Patients with Meth-ICH were more often younger (51.2 vs. 62.2 years, *p* < 0.001), male (77.0% vs. 61.4.0%, *p* < 0.05), and smokers (44.3% vs. 13.4%, *p* < 0.001). Non-Meth-ICH was more likely to have history of hypertension (72.61% v. 59%, *p* < 0.05) or antithrombotic use (10.9% vs. 1.6%, *p* < *0.05*). There was no significant difference in clinical severity, hospital length of stay (LOS), rate of functional independence (29.5% vs. 25.7%, *p* = 0.534), or mortality (18.0% vs. 24.6%, *p* = 0.267) between the 2 groups. Methamphetamine use was not an independent predictor of poor outcome. Despite difference in demographics, Meth-ICH is similar to Non-Meth ICH in hospital course and outcome.

## Introduction

Spontaneous intracerebral hemorrhage (ICH) is a devastating type of strokes^1,2^. The reported median 30-day mortality ranges from 17% to 40%, with rates of functional independency between 12% and 39%^1^. Recent randomized controlled studies have shown no significant benefit from intensive blood pressure control and early surgical intervention^3–5^. Since spontaneous ICH is potentially preventable, it is therefore a research priority to investigate any emerging risk of the disease.

Methamphetamine use is a serious public health crisis, with estimated 37 million active users and 2.6 million disability-adjusted life years lost in 2010^6,7^. Caplan at al. initially described incidence of intracerebral and subarachnoid hemorrhage from methamphetamine abuse^8,9^. Ischemic stroke was subsequently reported to be associated with methamphetamine use as well^10–14^.

Both population-based study and forensic analysis of fatal strokes showed significant predominance of hemorrhagic strokes in young methamphetamine users^15,16^. Methamphetamine use was associated with approximately fivefold increase in hemorrhagic stroke, twice higher than the risk from either cocaine or tobacco^15^. Lappin *et al*. performed a systemic review of methamphetamine-related stroke in 2017 and identified 81 hemorrhagic and 17 ischemic stroke cases from case reports and single center series^17^. Both types of stroke were approximately twice as common in males.

There appears to be an alarming trend of increasing prevalence of methamphetamine-associated ICH (Meth-ICH) in the regions around the Pacific rim^17^. Ho *et al*. initially described 11 patients with Meth-ICH at University of California San Francisco Medical Center between 2003 and 2007^13^. Nakagawa *et al*. then identified 25 patients with Meth-ICH (13%) at Queen’s Medical Center in Hawaii between July 2011 and January 2014^18^. Most recently, Swor *et al*. reported 41 Meth-ICH (16.4%) at University of California Davis Medical Center between January 2013 and December 2016^19^. Although Meth-ICH was increasingly reported in younger adults, there were conflicting data on hospital course and outcome^17–20^.

As the only academic Comprehensive Stroke Center in Orange County, California, we have witnessed significant increase in ICH volume in the last 5 years. The objective of this study was to investigate the demographics, hospital course, and outcome of Meth-ICH in comparison to Non-Meth-ICH.

## Methods

This is a retrospective observational study. It was approved by the University of California Irvine Institutional Review Board (IRB) and the Ethics Committee. Informed consents were waived as part of the IRB approval. All methods in the study were performed in accordance with the relevant guidelines and regulations.

Consecutive patients with spontaneous ICH at the University of California Irvine Comprehensive Stroke Center between January 1, 2011 and December 31, 2017 were identified by searching electronic medical records and the prospectively maintained American Heart Association (AHA)-*Get With The Guidelines (GWTG)-Stroke Registry*. ICH from cerebral aneurysm, arteriovenous malformation, brain tumors, coagulopathy, or trauma were excluded. Patients with Meth-ICH were identified by recorded history of methamphetamine use or a positive urine drug screen (UDS) at the time of admission. The UDS was performed using EMIT II Plus Amphetamines assay (1,000 ng/mL cutoff) with a sensitivity and specificity of 94.3% and 93.3%, respectively^21^. Those with a positive UDS while taking trazodone, Adderall, bupropion, or labetalol within 2 weeks of admission were excluded due to potential false-positive result^22^. Patients with no history of methamphetamine use and a negative UDS were included in Non-Meth-ICH group. Patients who denied history of drug abuse but had no UDS were excluded from the comparison analysis. All ICH patients were initially managed in the dedicated Neuroscience ICU with standard ICH order-set and clinical pathway by board-certified neurointensivists.

The following information was abstracted from chart review and the AHA *GWTG-Stroke Registry*: age, gender, race, past medical history, the highest blood pressure (BP) levels within 24 hours of admission, baseline National Institutes of Health Stroke Scale (NIHSS) score, Glasgow Coma Scale (GCS) score in ED, home medications, ICH location, intraventricular hemorrhage (IVH), ICH score, laboratory and imaging results, surgical interventions, ventilator support, length of stay (LOS) in the intensive care unit (ICU) and hospital, and modified Rankin Scale (mRS) at hospital discharge.

ICH location was categorized into deep (basal ganglia or thalamus), lobar, brainstem, cerebellum, multifocal or primary IVH^23^. ICH score was calculated as previously described^24^. Functional outcome was estimated per mRS. Since patients with mRS 3–5 could improve within 3–6 months after ICH, functional independence (mRS 0–2) and mortality (mRS 6) at hospital discharge were used as endpoints to explore the factors associated with outcomes.

### Statistical analysis

Continuous variables were described by mean ± standard deviation (SD) or median with interquartile range (IQR) based on the results of normality testing. Categorical variables were expressed by counts with percentages. Baseline characteristics and outcomes at discharge were compared between Meth-ICH and Non-Meth-ICH groups by Wilcoxon rank-sum test for continuous variables and χ2 test for categorical variables. Univariate analyses were performed initially to assess the possible factors associated with outcomes (functional independence and mortality at discharge). The cutoff value in univariate analysis for inclusion in the multivariate logistic regression was *p* < 0.1. Analyses were performed using SPSS software (version 23.0). A 2-tailed value of *p* < 0.05 was considered statistically significant.

## Results

### Demographics of patients with Meth-ICH and Non-Meth-ICH

**Figure 1:**
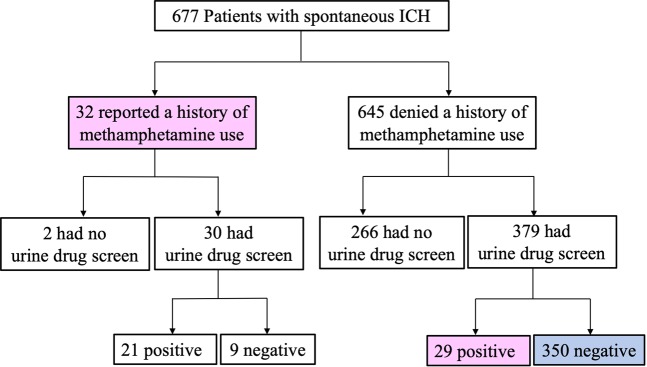
Flow chart for patient selection. Meth-ICH: reported a history methamphetamine use or had a positive urine drug screen (marked in pink); Non-Meth-ICH: denied a history of methamphetamine use and had a negative urine drug screen (marked in blue).

A total of 677 consecutive patients with spontaneous ICH between January 1, 2011 and December 31, 2017 were included. The flow chart for identification of Meth-ICH and Non-Meth-ICH is shown in Fig. 1. Thirty-two patients had recorded history of methamphetamine use. Among them, 30 had a UDS and 21 (70%) tested positive, indicative of methamphetamine use within the last 3 days^21^. Of the 645 patients who denied history of methamphetamine use, 379 (58.8%) had a UDS and 29 of them (7.7%) tested positive for amphetamine. Therefore, a total of 61 patients in the entire cohort (9%) were identified to have Meth-ICH (highlighted in pink color in Fig. 1). Given the fact that 7.7% of the patients who denied history of methamphetamine use had a positive UDS, the rate of Meth-ICH could be higher if all patients had a UDS.

As highlighted in blue color in Fig. 1, the 350 patients who denied a history of methamphetamine use and also had a negative UDS were classified as Non-Meth-ICH.

**Figure 2:**
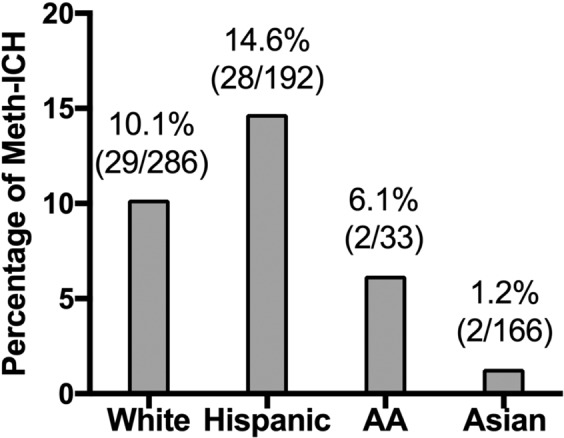
Racial disparities in the prevalence of Meth-ICH.

The mean age of the cohort was 63.4 ± 15.9 (range 18–100). Three hundred ninety-eight patients (58.8%) were male. There were 286 White (42.2%), 192 Hispanic (28.4%), 166 Asian (24.5%), and 33 African-American (4.9%) patients (Fig. 2). The rate of Meth-ICH was significantly higher in Hispanics (14.6% vs 1.2%; OR = 14.29, 95% CI 2.70–50.00; *p* < 0.001) and Whites (10.1% vs 1.2%; OR = 9.09, 95% CI 2.17–33.33; *p* < 0.001) than in Asians.

The characteristics of the patients with Meth-ICH or Non-Meth-ICH are summarized in Table 1. Compared with Non-Meth-ICH group, the patients with Meth-ICH were significantly younger (51.2 + 7.5 vs 62.2 + 15.9; *p* < 0.001) and more likely to be male (77% vs 61.4%; *p* = 0.019) and smokers (44.3% vs 13.4%, *p* < 0.001). Non-Meth-ICH was more likely to have history of hypertension (72.61% v. 59%, *p* < 0.05) or antithrombotic use (10.9% vs. 1.6%, *p* < *0.05*). There was no significant difference in highest systolic blood pressure (SBP) or diastolic blood pressure (DBP) within 24 hours of admission, NIHSS, GCS or ICH score between the 2 groups.

**Table 1:** Characteristics of patients with Meth-ICH or Non-Meth-ICH.

Variables	Meth-ICH (n = 61)	Non-Meth-ICH (n = 350)	*p*
Age	51.2 ± 7.5	62.2 ± 15.9	**<0.001**
Male	47 (77.0)	215 (61.4)	**0.019**
**Past medical history**			
Hypertension	36 (59.0)	254 (72.6)	**0.032**
Diabetes	9 (14.8)	83 (23.7)	0.121
Hyperlipidemia	7 (11.5)	70 (20.0)	0.115
Cardiac diseases	3 (4.9)	37 (10.6)	0.169
Antithrombotic agents	1 (1.6)	38 (10.9)	**0.023**
Smoking	27 (44.3)	47 (13.4)	**<0.001**
SBP, mmHg^a^	180.9 ± 41.5	181.2 ± 40.6	0.711
DBP, mmHg^a^	102.5 ± 24.1	99.5 ± 23.9	0.678
NIHSS	19 (6, 27)	12 (2, 22)	0.065
GCS	10 (4,16)	14 (10, 18)	0.084
ICH location			0.001
Deep	35 (57.4)	163 (46.6)	0.119
Lobar	10 (16.4)	133 (38.0)	**0.001**
Brainstem	8 (13.1)	26 (7.4)	0.137
Cerebellum	3 (4.9)	22 (6.3)	0.783
Primary IVH	5 (8.2)	5 (1.4)	0.009
Multifocal	0 (0)	1 (0.3)	1.000
IVH	35 (57.4)	167 (47.7)	0.164
ICH score			0.911
0	16 (26.2)	94 (26.9)	
1	15 (24.6)	93 (26.6)	
2	9 (14.8)	49 (14.0)	
3	11 (18.0)	51 (14.6)	
4	9 (14.8)	45 (12.9)	
5	1 (1.6)	16 (4.6)	
6	0 (0)	2 (0.6)	
Ventilator support	28 (45.9)	157 (45.0)	0.894
Surgical intervention	27 (44.3)	103 (29.4)	**0.022**
ICU LOS (days)	6 ± 6	7 ± 6	0.903
Hospital LOS (days)	14 ± 14	11 ± 10	0.056
mRS at discharge			0.328
mRS 0–2	18 (29.5)	90 (25.7)	0.534
mRS 3–4	23 (37.7)	102 (29.2)	
mRS 5	9 (14.8)	72 (20.6)	
mRS 6 (Death)	11 (18.0)	86 (24.6)	0.267

Abbreviations: SBP, systolic blood pressure; DBP, diastolic blood pressure;. NIHSS, National Institutes of Health Stroke Scale; GCS, Glasgow Coma Scale;. ICH, intracerebral hemorrhage; IVH, intraventricular hemorrhage;. ICU, Intensive Care Unit; mRS, modified Rankin Scale; LOS, length of stay. ^a^Highest SBP/DBP within 24 hours of admission. Data are n (%), mean ± SD, or median (interquartile range, IQR).

There was significant difference in ICH location between the 2 groups (*p* = 0.001), with Meth-ICH group having less lobar hemorrhage (OR = 0.32, 95%CI 0.16–0.65, *p* = 0.001) and more primary IVH (OR = 6.16, 95%CI 1.73–21.97, *p* = 0.009).

Although Meth-ICH was more often treated with surgical interventions, likely due to younger ages, there was no significant difference in requirement for ventilator support and LOS in the ICU or hospital between the 2 groups.

There was also no significant difference in either functional independence (OR = 1.21, 95% CI 0.66–2.20; *p* = 0.534) or mortality rate at hospital discharge (OR = 0.68, 95% CI 0.34–1.36; *p* = 0.267). Methamphetamine use *per se* was not a predictor of poor outcomes in patients with ICH.

### Predictors of outcomes

Factors associated with functional independency and mortality at hospital discharge were analyzed using univariate and multivariate models. Overall, lower NIHSS and higher GCS score were associated with functional independence at hospital discharge per multivariate analysis (Table 2). Higher NIHSS, ICH score, ventilator support, and absence of surgical interventions were independent predictors of mortality (Table 3).

**Table 2:** Factors associated with functional independence at discharge.

Variables	Functional independence (n = 108)		
	Univariate analysis	Multivariate analysis	*p*
	*p*	OR (95% CI)	
Age	0.486		
Male	0.462		
Race	0.304		
HTN	**0.076**	0.54 (0.28–1.05)	0.07
DM	0.164		
Smoking	**0.002**	1.35 (0.67–2.71)	0.405
Antithrombotic agents	0.390		
Meth use	0.534		
ICH location	0.394		
SBP	0.515		
DBP	0.257		
NIHSS	< **0.001**	0.73 (0.67–0.80)	**<0.001**
GCS	< **0.001**	1.29 (1.07–1.56)	**0.008**
ICH score	**<0.001**	0.89 (0.56–1.30)	0.454
Glucose	0.332		
Creatinine	0.583		
Surgical interventions	**<0.001**	0.88 (0.29–2.68)	0.821
Ventilator support	**<0.001**	0.41 (0.13–1.29)	0.128

Abbreviations: SBP, systolic blood pressure; DBP, diastolic blood pressure; NIHSS. National Institutes of Health Stroke Scale; GCS, Glasgow Coma Scale. ICH, intracerebral hemorrhage; mRS, modified Rankin Scale.

**Table 3:** Factors associated with hospital mortality.

Variables	Hospital mortality (n = 97)		
	Univariate analysis	Multivariate analysis	*p*
	*p*	OR (95% CI)	
Age	0.107		
Male	0.354		
Race	0.137		
HTN	0.245		
DM	**0.004**	1.58 (0.70–3.55)	0.272
Smoking	**0.051**	0.97 (0.34–2.75)	0.948
Antithrombotic agents	0.935		
Meth use	0.267		
ICH location	**<0.001**	1.07 (0.78–1.47)	0.679
SBP	**0.047**	1.01 (0.99–1.02)	0.237
DBP	**0.070**	0.99 (0.97–1.02)	0.513
NIHSS	<**0.001**	1.09 (1.02–1.17)	**0.013**
GCS	<**0.001**	0.90 (0.78–1.04)	0.169
ICH score	**<0.001**	2.67 (1.80–3.97)	**<0.001**
Glucose	**0.003**	1.00 (0.99–1.01)	0.856
Creatinine	0.134		
Surgical interventions	**0.068**	0.24 (0.11–0.51)	**<0.001**
Ventilator support	**<0.001**	3.95 (1.37–11.4)	**0.011**

Abbreviations: SBP, systolic blood pressure; DBP, diastolic blood pressure. NIHSS, National Institutes of Health Stroke Scale; GCS, Glasgow Coma Scale. ICH, intracerebral hemorrhage.

## Discussion

This study represents the largest single center cohort of Meth-ICH yet reported. It demonstrates that methamphetamine is an important risk factor for ICH in young males, smokers, Hispanic and White populations in Southern California. The rate of Meth-ICH was 9% in our large cohort, as compared to 13% and 16.4% reported previously^18,19^. Of note, of the 645 patients who denied history of methamphetamine use, only 379 patients (58.8%) had a UDS and 29 (7.7%) were urine positive for amphetamine. The actual rate of Meth-ICH could be significantly higher if all ICH patients had a UDS.

The observed age disparities in patients with Meth-ICH versus Non-Meth-ICH were consistent with previous reports^18,19^.

Although ordering UDS at admission was routine practice at our Stroke Center, the decision to obtain the test was at the discretion of the ED and on-call stroke team. Previous report showed ethnic disparities in ordering drug screens in patient with ICH, with young African Americans having more UDS^25^. We examined the ordering of drug screens for ICH patients between 2013 and 2017^26^. Of the 596 ICH patients, 357 (60%) had a UDS. A UDS was more likely to be obtained in patients who were younger (age < 45), male, smokers, self-reported methamphetamine users, with more severe deficit at presentation (NIHSS > 4), without diabetes, or not taking anticoagulant. There was no significant difference in UDS among different races (*p* = 0.319)^26^. Therefore, the higher rates of Meth-ICH in Hispanics and Whites were not due to bias in ordering UDS. In addition, hypertensive ICH is very prevalent in Asians^2^. However, only 1.2% (2/166) Asians in this cohort had Meth-ICH. These findings may have significant public health implication and may guide targeted community education for the prevention of Meth-ICH.

The majority of Meth-ICH was located in the basal ganglia/thalamus, suggesting deep white matter small-vessel injuries from direct toxicity of methamphetamine or increased sympathetic system activation^15,17,27^. The proportion of lobar hemorrhage was significantly higher in Non-Meth-ICH (38.0% vs 16.4%), likely due to older age and history of hypertension, ischemic stroke, or cerebral amyloid angiopathy^28–30^.

In our current study, there was no significant difference in ICH severity, ventilator support, LOS in the ICU or hospital, rate of functional independence or mortality at hospital discharge between Meth-ICH and Non-Meth-ICH. The severity of neurologic deficits at admission was the main predictor of functional independence and mortality. Methamphetamine use and demographic features including age were not independently associated with outcomes as they had been in previous studies^17,29,30^.

Of note, our findings are significantly different from what was previously reported. Swor *et al*. showed that patients with Meth-ICH had significantly longer LOS in the ICU (10 ± 8 vs 5 ± 5 days) and hospital (18 ± 27 vs 8 ± 8 days) than Non-Meth-ICH^19^. They also showed higher mortality rates (29% vs 34%), as compared to our study (18% vs 25%). Several factors may account for lack of difference in LOS between the two groups and lower mortality rates in our study. First, although the Meth-ICH patients had higher NIHSS score, they were younger with less comorbidities as compared with Non-Meth-ICH patients. Second, there was no significant difference in highest blood pressures, GCS, ICH score and ventilator support between Meth-ICH and Non-Meth-ICH groups. Third, all ICH patients were initially managed in the dedicated Neuroscience ICU with standard ICH order-set and clinical pathway by board-certified neurointensivits at our center. Management of ICH by neurocritical care specialists have been shown to be associated with shorter LOS and reduced mortality^31–33^.

The mechanisms by which methamphetamine may cause ICH remains unclear. In 1970, Citron *et al*. studied 14 patients with polysubstance abuse, with almost all admitting methamphetamine use, and found necrotizing angiitis in medium-sized and small arteries of the brain and other organs^34^. Rumbaugh *et al*. analyzed the angiographic features of patients with methamphetamine abuse and described beaded arteries, segmental changes in vessel caliber, and regions of slow flow^35^. In monkeys receiving intravenous methamphetamine for two weeks, angiography showed similar beading and segmental changes, and necropsy revealed small cerebral hemorrhages, zones of infarction, and microaneurysms^36^. Davis *et al*. reported 4.9% incidence of methamphetamine intoxication in all autopsies in San Diego County from 1 January 1987 to 31 December 1993 and suggested possible propagation and rupture of berry aneurysms from methamphetamine use^37^. Of note, methamphetamine is a potent sympathomimetic. ICH may also occur secondary to methamphetamine-induced hypertensive surge in the absence of pre-existing cerebrovascular disease^38^. Chronic use of methamphetamine may cause long-term systemic hypertension and vessel damage^13,17,27,39^. Further studies are warranted to investigate the mechanisms of Meth-ICH.

To the best of our knowledge, this is the largest single-center cohort to compare the clinical characteristics and outcomes of patients with Meth-ICH versus Non-Meth-ICH. Our study demonstrates that there is no significant difference in severity of ICH, LOS, favorable outcome and mortality between Meth-ICH and Non-Meth-ICH. In addition, we found considerable proportion of patients who denied history of methamphetamine use turned out to have positive urine drug test, indicating possible higher prevalence of methamphetamine use in patients with ICH. The strengths of this study are the comprehensive comparison of the clinical features and outcomes of Meth-ICH verse non-Meth-ICH. Our findings provide better understanding of Meth-ICH vs Non-Meth-ICH and may help develop strategies for the effective treatment and prevention of Meth-ICH.

Our study has a few limitations. First, this is a single-center study performed in Southern California. Our findings may not be generalizable to other regions or populations. Second, despite denying drug use history and negative urine drug test, it is still possible that there were undetected meth users in the Non-Meth-ICH group. The relatively small sample size in the Meth-ICH group and possible undetected meth users in the Non-Meth ICH group may contribute to the lack of difference in hospital course and mortality between the two groups. Studies with large sample size will be needed in the future to validate our findings. Third, in this retrospective study, there was no information about the route (inhalation, ingestion or injection), frequency, and duration of methamphetamine use in most patients. The temporal relationship between recent methamphetamine use and ICH onset could not be established. In addition, UDS is only sensitive in detecting amphetamines within 4 days of recent use and may not identify chronic users. Lastly, patients may have history of polysubstance abuse. Of note, cocaine and excessive alcohol use are also risk factor for ICH^40,41^. Cocaine was only detected in 2 of the 50 patients with UDS-confirmed methamphetamine use and could not be significant compounding factor in our cohort. However, we had no data on excessive alcohol use in our case series.

In summary, methamphetamine use is an emerging risk factor for ICH in young males, smokers, Hispanic and White populations in Southern California. There is no significant difference in ICH severity, LOS in the ICU or hospital, functional independence or mortality rate between Meth-ICH and Non-Meth-ICH. Targeted public and community education may be essential for the prevention of Meth-ICH.
